# Methodology for a Series of Rapid Reviews on Virtual Care in Rehabilitation, Reviewing Its Advantages and Challenges to Inform Best Practices

**DOI:** 10.3390/clinpract14060214

**Published:** 2024-12-13

**Authors:** Jennifer Sigouin, Anne Hudon, Mirella Veras, Simon Beaulieu-Bonneau, Sabrina Cavallo, Dahlia Kairy

**Affiliations:** 1 Institut Universitaire sur la Réadaptation en Déficience Physique de Montréal, Centre de Recherche Interdisciplinaire en Réadaptation du Montréal Métropolitain (CRIR), Montréal, QC H3S 1M9, Canada; jennifer.sigouin@mail.mcgill.ca (J.S.); sabrina.cavallo@umontreal.ca (S.C.); 2 École de Réadaptation, Faculté de Médecine, Université de Montréal, Montréal, QC H3S 1M9, Canada; 3 Department of Physical Therapy, College of Rehabilitation Sciences, Rady Faculty of Health Sciences, University of Manitoba, Winnipeg, MB R3E 0T6, Canada; mirella.veras@umanitoba.ca; 4 École de Psychologie, Université Laval, Québec, QC G1V 0A6, Canada; simon.beaulieu-bonneau@psy.ulaval.ca

**Keywords:** telehealth, telerehabilitation, e-health, digital health, protocol, methodology, ethics, equity, rapid review

## Abstract

**Background/Objective**: Over the past two decades, the utilization of virtual care in rehabilitation has witnessed a significant surge; this is owing to the widespread availability of technological tools and the global impact of the COVID-19 pandemic. As a result, discussions surrounding the relevance and benefits of telerehabilitation have gained prominence among practitioners, who continually seek to enhance patient care while maintaining high standards of quality. Associated with these discussions are concerns over being able to provide care in an ethical way, as well as addressing equity issues that might be hindered or improved via telerehabilitation. To address the ethical and equity concerns around telerehabilitation, a series of five parallel rapid reviews of the scientific literature were conducted, focusing on different rehabilitation fields: physiotherapy and occupational therapy (1); speech therapy and audiology (2); psychology and neuropsychology (3); and in two age groups: older adults (4); and pediatrics and young adults (5). The objective of this series of rapid reviews is to evaluate the evidence presented regarding telerehabilitation; identifying and recommending best practices especially in the realm of ethics and equity. **Methods:** Medline; CINAHL; and EMBASE were searched between 2010 and 2023 for English or French-language reviews (2010–2020) and individual studies (2020–2023) pertaining to telerehabilitation and these fields of interest. Data were extracted following a standardized form focusing on: outcomes; findings; quality assessment/biases; limitations; and discussion of ethical and equity concerns. **Results:** The results are presented according to the most relevant themes, which include: findings; strengths; limitations; and ethical/equity considerations. **Conclusions:** This research presents a methodology rarely published before, on how to conduct multiple parallel rapid reviews on the theme of telerehabilitation, based on different rehabilitation fields and age groups. This research will shape future guidelines and standards in applying ethical and equity standards in telerehabilitation.

## 1. Introduction

Virtual care in rehabilitation, also known as telerehabilitation (TR), encompasses the delivery of rehabilitation services through various technologies such as videoconferencing, audio, chat/text, websites, recorded videos or images, and e-visits, among others [1,2]. Over the past two decades, the utilization of virtual care in rehabilitation has seen a significant surge, owing to the widespread availability of technological tools (e.g., smartphones, tablets) and the global impact of the COVID-19 pandemic, which accelerated the demand and integration of virtual care options [3]. During the COVID-19 pandemic, physical interactions were constrained, while the demand for rehabilitation services persisted, and in some cases, even increased [4]. Consequently, the necessity to offer rehabilitation services through virtual care became paramount, highlighting the potential of telerehabilitation to provide continuity of care across diverse contexts. Fortuitously, technological advancements made it relatively feasible to provide such virtual services, at least for a portion of the patient population. As a result, discussions surrounding the relevance and benefits of telerehabilitation have gained prominence among practitioners, who continually seek to enhance patient care while maintaining high standards of quality.

Technological advancements have facilitated the delivery of these services, but the rapid growth in telerehabilitation has brought complex ethical and equity considerations to the forefront. For many patients, telerehabilitation has proven as effective as in-person rehabilitation [5,6,7], particularly when catering to remote and underserved populations enabling greater access to care where physical proximity to clinics may be limited [8,9,10]. Despite these positive findings, certain practitioners and clinical settings have shown reluctance to adopt telerehabilitation, expressing concerns about its ethical application and questioning its efficacy [11,12], especially for specific groups. For example, patients with limited access to or knowledge of digital tools may struggle to fully benefit from telerehabilitation, amplifying disparities, especially in vulnerable populations [13,14]. Hence, it is imperative for the field of rehabilitation to critically examine the use of telerehabilitation, identifying the contexts where it is most suitable and how it can be effectively used to uphold quality care, and to address equity and ethical considerations. Indeed, the rapid growth of telerehabilitation implementation mostly due to the COVID-19 pandemic has raised apprehensions among practitioners who fear that ethical standards might not be adequately established and implemented [15,16]. Additionally, there is a growing recognition of the need to address equity issues when deploying telerehabilitation services as accessibility to virtual care can vary widely across different populations [17].

Rehabilitation professionals and patients can still be largely unfamiliar with new technologies which can raise ethical concerns regarding informed consent, information sharing, safety and confidentiality, just to name a few [18]. As telerehabilitation adoption grows, it is essential for the field to examine not only the contexts in which these services are most suitable but also the ways in which they can be implemented to uphold high standards of ethical and equitable care. Ethical and equitable standards must address these challenges to ensure that telerehabilitation is both safe and accessible, and that patients receive quality care without compromising their rights or safety. Furthermore, accessibility to virtual care can be limited, which can accentuate already existing inequities in the patient population, especially for those who are already in a vulnerable position [19,20]. For example, remote populations could benefit from virtual care services, but only if the technology is accessible to them (and of good quality), and they know how to use it [21,22,23].

Therefore, it is important for clinicians to be well informed about ethical and equity considerations when it comes to offering services via telerehabilitation. Clinical ethics—the application of ethical theories, principles, rules, and guidelines to clinical situations [24]—focuses on four main principles of ethics; including beneficence, nonmaleficence, autonomy, and justice [25]. Beneficence and nonmaleficence highlight the importance of keeping the patient’s best interest as the primary objective of any clinical practice. For example, one must pay greater attention to adverse events or potential risks, ensuring that patients are not at an increased risk of injuring themselves. This involves closely monitoring for signs of discomfort, fatigue, or improper technique during interventions, and making timely adjustments to the treatment plan as needed. This is particularly important for patients who receive virtual care in rehabilitation, as they are often required to be actively engaged in their care compared to other contexts [26]. Related to this is the notion of autonomy, where patients should be allowed to exercise their ability to self-determination by making their own choices regarding their treatment [27]. Finally, justice refers to the ability to offer services to everyone who needs them, making sure that people with disabilities or people suffering from other disadvantages (e.g., social class) are not being excluded from receiving those services [28]. The intersection of ethics and equity in telerehabilitation is not only about delivering care, but about doing so in ways that enhance access and support fairness across diverse populations. If telerehabilitation is implemented with these considerations in mind, it holds the potential to reduce health inequities and provide meaningful support to those who might otherwise be underserved.

In order to provide meaningful support, different considerations have to be examined for different populations. For example, many any older adults may have limited familiarity with digital technology or face physical challenges, such as reduced vision or hearing, which can affect their ability to engage with telerehabilitation effectively. Ethical considerations for this group include ensuring user-friendly interfaces, providing adequate support, and maintaining clear communication to support their autonomy and consent processes. Telerehabilitation can offer older adults the convenience of receiving care from home, reducing the need for transportation, which may be challenging for those with mobility issues [29].

Similarly, rural and Indigenous populations may face challenges due to unreliable internet connectivity or limited access to digital devices, which can impede their participation in virtual care. Ensuring equitable access requires targeted investment in infrastructure and culturally sensitive care models that respect the unique needs of these communities. By bringing rehabilitation services to remote areas, telerehabilitation can help bridge the gap in access to specialized care; supporting continuity of care and promoting better health outcomes in communities with limited healthcare resources [30]. It also has the potential to empower Indigenous communities by increasing their autonomy through clinician and patient engagement, and their trust in the care process [31].

In addition, individuals with physical or cognitive disabilities may encounter obstacles in using telerehabilitation platforms designed for general populations. Ethical practice requires adaptations to interfaces, tailored accessibility features, and support for caregivers to ensure that patients can engage with care safely and independently. Telerehabilitation, when adapted for accessibility, can empower individuals with disabilities by enabling greater autonomy in their care, offering flexible and personalized treatment options [16].

For patients in low-income households, the affordability of digital devices and internet services can be a barrier to accessing telerehabilitation. Equity considerations for these populations involve addressing financial barriers, providing low-cost or subsidized services, and exploring community-based solutions to ensure access to care. Telerehabilitation has the potential to reduce healthcare costs for low-income patients by minimizing travel expenses and time away from work, making care more accessible and sustainable. [32].

In addition, language barriers may hinder non-English-speaking patients from fully understanding and benefiting from telerehabilitation. Providing multilingual support and culturally adapted materials is essential to ensure they can engage in treatment, while respecting their autonomy and cultural background. Multilingual and culturally sensitive telerehabilitation services can make care more inclusive, allowing non-English-speaking populations to participate more fully in their treatment and make informed decisions about their health [33].

Despite these challenges, telerehabilitation holds significant promise in improving accessibility, continuity, and quality of care for all people with disability. By bridging geographical gaps and providing flexible options for participation, telerehabilitation can help reduce barriers to care for those who have historically been underserved. For example, older adults and rural residents who may have difficulty traveling to in-person appointments, benefit greatly from the convenience of virtual sessions, which could allow them to engage in rehabilitation from home [29]. Likewise, multilingual support and culturally tailored services can improve engagement and health outcomes for diverse communities [33].

When implemented with ethical and equitable considerations in mind, telerehabilitation has the potential to enhance the healthcare experience for patients by reducing physical and logistical barriers, increasing patient-centered care options, and supporting a more inclusive and accessible healthcare system. By addressing these unique needs proactively, telerehabilitation can play a transformative role in expanding the reach and inclusivity of rehabilitation services, helping to close gaps in health access and equity.

In order for telerehabilitation’s full potential to be reached, given the large amount of literature that is rapidly evolving, clinicians require expedient access to synthesized evidence on the best practices of telerehabilitation to ensure its ethical and equitable implementation. This paper aims to address these issues by describing the protocol used to conduct a series of parallel rapid reviews, whose objectives consist of providing a comprehensive review of the current state of telerehabilitation and offering evidence-based recommendations for its optimal utilization, considering ethical and equity issues. By doing so, we hope that our rapid reviews will facilitate informed decision-making among practitioners and contribute to the advancement of virtual care in the field of rehabilitation.

Rapid reviews were chosen as an efficient method to gather and summarize relevant information on the topic, employing a consistent methodology, while allowing some flexibility not typically possible in systematic reviews [34]. Opting for rapid reviews allows the research team to complete the review within a shorter timeframe and focus on specific aspects of the literature deemed most pertinent, such as the types of telerehabilitation used or a specific patient group [35].

Due to the project’s complexity and scope, separate parallel rapid reviews were conducted, each focusing on different rehabilitation fields and/or populations of interest. We chose to approach different professions because, while certain issues may be unique to each context, insights or issues identified in one area might be applicable to and inform others. Many professions share commonalities, and in the field of rehabilitation, multidisciplinary and interdisciplinary settings are common. The lessons learned in this shared environment can be particularly valuable; as presented in this protocol which describes the process used to conduct the reviews. This research presents the methodology put in place to address the complexity of conducting multiple parallel rapid reviews on the theme of telerehabilitation across patient populations and rehabilitation contexts, with a common focus on ethical and equity considerations.

This protocol offers valuable insights into the unique process that is involved in conducting parallel rapid reviews on the large evolving topic that is telerehabilitation, which can help other researchers who would like to pursue similar research avenues. To our knowledge, very few articles have conducted and described such methodology before us, especially in the realm of telerehabilitation. Moreover, the findings from the individual reviews as well as the common findings will serve as a valuable resource, presenting best practices regarding telerehabilitation to guide its implementation and usage in clinical settings, and inform decision makers. By synthesizing, comparing, contrasting and presenting this evidence, we aim to contribute to the advancement of ethical and equitable virtual care practice in the field of telerehabilitation.

## 2. Materials and Methods

### 2.1. Study Design

This protocol presents the methodology developed for a series of parallel rapid reviews of the scientific literature regarding telerehabilitation. The methodology for the individual rapid reviews is guided by the Cochrane Rapid Reviews Interim Guidance from the Cochrane Rapid Reviews Method Group (2020) [36], which is defined as a “form of knowledge synthesis that accelerates the process of undertaking a traditional systematic review through streamlining or omitting specific methods to produce evidence for stakeholders in a resource-efficient manner” (p. 14). This study is part of a larger research project titled “Avoiding pitfalls in virtual care: paving the road for more ethical and equitable policies and practices in rehabilitation”, and encompasses a rapid review (Phase I) of scientific literature to identify current clinical and organizational best practices in virtual rehabilitation. The study is registered under Prospero ID: CRD42020207602.

The series of rapid reviews is being conducted to examine virtual care in various rehabilitation settings and different patient groups encompassing motor, sensory, auditory, and cognitive deficits. Specifically, the reviews focus on the following rehabilitation professions: physiotherapy, occupational therapy, speech therapy, audiology, and psychology/neuropsychology. These professions were chosen because they have established professional orders, facilitating the identification of best practices in their respective fields and increasing the likelihood of research findings influencing the guidelines offered by these professional orders. The objective of this study is to present the methodology used across the rapid reviews, the challenges faced and the strategies put in place to ensure a level of cohesiveness across the reviews.

In addition, this protocol describes our process in conducting a series of parallel rapid reviews which requires additional methodological considerations. This protocol will provide valuable insights regarding the methodological implications involved in this process of conducting a series of parallel rapid reviews at the same time. Given that each review focuses on a specific rehabilitation profession and/or population, each rapid review is tailored so as to capture the specificities of the fields and provide important information regarding the current usage of telerehabilitation as well as its strengths and weaknesses in each area.

Five rapid reviews centered on the following themes were planned:

1. Physiotherapy and occupational therapy (includes adults aged 18 and over)
2. Speech therapy and audiology (includes adults aged 18 and over)
3. Psychology and neuropsychology (includes adults aged 18 and over)
4. Older adults (aged 50 and over)
5. Pediatrics (0–18) and young adults (aged 18–25)

### 2.2. Study Team Structure

Separate teams are assembled for each rapid review. Team members are accompanied by a coordinator, who ensures consistency between groups while allowing flexibility to account for each domain’s unique realities. This allows lessons learned and strategies developed to be shared between reviews in a timely manner. The team is composed of individuals with both clinical and research backgrounds, ensuring methodological rigor and clinically relevant perspectives throughout the process. The coordinator keeps track of the team’s decisions and maintain a cohesive approach throughout the project.

### 2.3. Search Strategy

Database search strategies have been developed for Medline, EMBASE, and CINAHL, combining keywords and MeSH terms for telerehabilitation and the professions of interest; namely, physiotherapy, speech therapy, occupational therapy, audiology, psychology and neuropsychology, or the relevant age group or topic of interest. Only articles written in English or French are retained and duplicates removed from the databases. The search terms are elaborated with a team of researchers and a librarian, in order to make sure to capture the most relevant publications; namely, those pertaining to the realm of rehabilitation related to motor, sensory, auditory, or cognitive deficits. For example, articles pertaining to psychologists helping patients in drug rehabilitation processes are excluded. The search was conducted in two phases:

(1) A first search strategy was conducted to identify only reviews of articles published between 2010 and 2020. The year 2010 was chosen as it represents the introduction of smartphones, which significantly improved the technology available for virtual services. Smartphones served as a catalyst for the development of various technological innovations in this domain. By choosing the year 2010 as the focal point, the research aimed to capture publications that had sufficient time to investigate and assess the influence of smartphones and other associated technologies and platforms on the progression of virtual care services. This timeframe allowed researchers to observe and analyze the evolving trends and advancements in the field, especially in relation to telehealth and telerehabilitation. The database was shared among our different teams to identify the articles that were relevant to their field of interest. Given the complexity of the task at hand and to respect the idea of the rapid review, we excluded reviews that did not clearly indicate the inclusion criteria in the full manuscript in order for our rapid reviews to be completed within a reasonable timeframe. For example, some reviews potentially pertaining to telerehabilitation and psychology/neuropsychology did not list clearly the professions included in their review, making it difficult to determine if these reviews were relevant or not, without having to read each individual article included in the reviews. We are aware that this could exclude relevant articles, but given that our focus is on the results presented in the reviews and not on the individual studies themselves, this strategy respected the goal of conducting rapid reviews. Unlike systematic reviews, which aim for exhaustive coverage of all relevant literature, a rapid review focuses on summarizing key findings, ensuring that the review process remains credible, even if it is not exhaustive [35]. In addition, although a rapid review is not exhaustive, the strategy we employed for information gathering is robust and captures a significant volume of relevant data. This enhances confidence that the findings will be reflective of the evidence on the topic.
(2) In order to capture the results of the most recent studies, we only included original studies from 2020, given the extent of the literature captured with the first search strategy. We did not include reviews after 2020, since recent reviews are unlikely to capture the latest articles and would likely repeat findings from earlier reviews. Given the substantial body of evidence included in reviews up to 2020, we felt it would be more relevant to capture original studies conducted in real-world contexts, including during the COVID-19 pandemic, which led to a rapid increased use of telerehabilitation. The pandemic significantly accelerated the adoption of telerehabilitation, resulting in a notable increase in the volume of related literature. Documenting this surge is crucial, as the rapid implementation of telerehabilitation during the pandemic may have been accompanied by reduced attention to ethical and equity considerations. This second search gave us a second database that we divided into different professions when applicable.

We kept the two databases (one with reviews, one with individual articles) separate to make sure that the data extraction phase allowed us to keep track of the potential differences between the two. For example, it is possible that the most recent publications offer a new focus on a facet of telerehabilitation that was not present before. Given the amount of academic literature found, grey literature is excluded at this time.

### 2.4. Study Selection

The criteria for inclusion are articles that study virtual care and include telerehabilitation as one of those types of care. It is not necessary for the article to solely focus on telerehabilitation in order to be included in our search, but articles that do so will be emphasized in our analyses. For the reviews of reviews, all types of reviews are included (e.g., systematic reviews, scoping reviews, rapid reviews) except those that solely present a literature review where their methodology, including the choice and analysis of the articles chosen, are not clearly reported. Abstracts, commentaries and letters to editors are excluded because they often lack detailed data, rigorous methodology, and comprehensive analysis. To ensure our analyses are based on robust evidence, we focused on full articles with clearly documented methods and thorough discussions. In this study, the rehabilitation fields include: physiotherapy, speech therapy, occupational therapy, audiology, psychology and neuropsychology. Any medium used to deliver telerehabilitation services via information and communication technology is included, including videoconferencing, email, apps (excluded if only used for self-monitoring with no input from a rehabilitation professional), web-based communication, and telephone communications. Articles that only mention telephone interventions are included, but will be examined separately in our analyses as our goal is to better inform current practices with newer technologies.

### 2.5. Data Collection and Extraction

Covidence or Rayyan software is used to screen and extract the data, depending on what team members had access to. The following stages are followed for each article using: (1) Title and abstract screening, (2) full text review, and (3) data extraction. For the title and abstract screening phase, articles that correspond to our inclusion criteria and those that do not have sufficient information to determine whether or not it meets the inclusion criteria are kept for full text review. The full text is then reviewed at the next stage to determine if they meet the inclusion criteria or not. For the full-text review, only articles that meet the inclusion criteria are included. Overall, the data collection is inspired by the Cochrane rapid reviews guidelines [36]. There are two reviewers for at least 10% of the articles to screen the titles, abstracts and full text, applying the inclusion and exclusion criteria at every phase. When there is agreement between the reviewers, only one reviewer conducts the rest of the review. When there is disagreement, conflict resolution is done between the two reviewers at first, followed by a second discussion with the team to make sure that the quality of reviewing and consistency is maintained. If the number of conflicts is minimal (less than 30%), we then move on to the next phase. If not, the second reviewer looks at an additional number of articles (between 10 and 20) and the resolution of conflicts follows the same procedure. The PICOT checklist (Table 1) guides the structure of the data collection and extraction. Two of the reviews utilized the PROGRESS-Plus framework and the Equitable Virtual Rehabilitation in the Metaverse Era framework to identify dimensions where potential inequities may exist within telerehabilitation interventions [37,38]. For each rapid review, at the full text stage, the data extraction is completed by one of two reviewers using a standardized form that was developed by several team members, with regular meetings with other team members to discuss the findings. The coordinator attends every meeting to receive feedback from each team regarding their experiences with the screening and improve the strategies based on their feedback. The standardized form can be tailored to the specificities of each review by adding certain elements, as well as the amount of details provided in the articles. The core data elements are discussed among each team member to decide which will be emphasized in the analyses, identifying the strengths and limitations of each article/review. Among the common elements identified in our data extraction are outcomes, findings, quality assessment/biases, limitations, discussion of ethical and/or equity considerations; in order to identify key strengths and limitations of telerehabilitation, as well as best practices, if identified. Given that we looked at a range of types of reviews, there is often insufficient information provided regarding their quality grading to apply an entire framework of quality assessment. We focused on two questions regarding quality assessment, that were inspired by the systematic review appraisal worksheet published by the Centre of Evidence-Based Medicine of the University of Oxford [39]: (a) were all relevant studies included? and (b) were the criteria used to select articles for inclusion and exclusion appropriate, or at risk of introducing a bias? We also took note if a review used a quality assessment method to evaluate the articles they included in their reviews. The study was conducted according to the guidelines of the Declaration of Helsinki, and approved by the Research Ethics Committee of Centre Intégré universitaire de santé et de services sociaux du Centre sud de l’île-de-Montréal on 15 September 2022. Ethical code: MP-50-2022-1610.

### 2.6. Data Analysis

For each rapid review, a research team is established and meets regularly to discuss the evidence and decide what should be emphasized in our analyses. The results are then presented according to the most relevant themes, which include: findings, strengths, limitations, ethical/equity considerations. The level of evidence provided by each article is assessed by looking at the amount and quality of information provided in each article. Emphasis on issues pertaining to ethics and equity is applied, paying attention to: beneficence, nonmaleficence, autonomy, and justice. More precisely, we focus on informed consent, information sharing, confidentiality, technological knowledge, access and quality, adverse events, a safe place to conduct sessions, equal access to care for vulnerable and marginalized populations.

## 3. Results

We expect to present results focused on the following elements: strengths, weaknesses, limitations/biases, ethical and equity considerations; with recommendations for best practice guidelines. As an example of a strength, we expect certain studies to mention how telerehabilitation can increase accessibility to services for certain groups, such as individuals living in rural areas, where finding in-person appointments could be difficult. Reduced transportation is another strength that we expect studies to explore, which can help reduce travelling time and help individuals with mobility issues. On the other hand, studies will likely mention the fear regarding the quality of services being reduced as well as findings regarding the impact on the therapeutic relationship with their clinicians. Additionally, the parallel reviews will provide a comprehensive overview of the current landscape of telerehabilitation, identifying gaps that require further research. For example, we expect that several studies will mention that the cost can be an impediment to the implementation of telerehabilitation, but that very few of them will have assessed directly its impact. Similarly, for characteristics such as gender and race, we expect that some studies will mention them as potential biases, but that a low number of studies would have directly included them in their analyses. Furthermore, we expect few studies to explicitly examine ethical and equity issues as their research objectives.

The findings will also explore various virtual care models, potential barriers to equitable access (e.g., cost, digital literacy, access to good internet access, just to name a few), and considerations for implementing these interventions in diverse populations such as the elderly, rural populations and marginalized ones. Furthermore, we will likely note differences in telerehabilitation usage across different occupations. For example, the field of speech-language pathology has been more proactive in integrating telerehabilitation into practice compared to physical therapy, where clinicians tend to be more hesitant. Many physiotherapists still believe that assessments need to be conducted in person, which might have slowed the adoption of telerehabilitation in their field. All of these findings will be particularly valuable for policymakers, contributing to decisions on resource allocation, regulatory frameworks, and strategies to ensure equitable and effective delivery of telerehabilitation services.

## 4. Discussion

The objective of this series of rapid reviews is to evaluate the evidence available regarding telerehabilitation, focusing on best practices especially in the realm of ethics and equity. This is particularly relevant in the current context where access to health services is becoming increasingly difficult. Telerehabilitation can been perceived as a potential solution to this lack of access, but must be implemented properly, upholding the best ethical and equity principles. In addition, the reviews are designed to present best-practice evidence within the field of telerehabilitation—more specifically for the physiotherapy, occupational therapy, speech therapy, audiology, psychology and neuropsychology professions—and to provide guidance for clinical implementation. It will likely highlight some similarities and differences between the disciplines, as well as where each one can learn from the others. The findings from these rapid reviews are intended to have a direct impact on clinical practice. Thus, by identifying best practices in telerehabilitation, especially in the context of ethics and equity, the reviews will help clinicians and policymakers make informed decisions about the integration and use of telerehabilitation across different disciplines.

### Study Limitations

Given the scope of the project, there may be differences between the rapid review teams. To address this, we have established a foundational research method designed to be as applicable and relevant as possible across the different teams while allowing adaptation to their specific needs. For instance, the Speech-Language Pathology team may focus on a younger population, such as children with developmental or communication disorders, who often present distinct physical and cognitive challenges. In contrast, the Physical Therapy and Occupational Therapy team may primarily address older adults or individuals with mobility or functional impairments, emphasizing rehabilitation strategies for improving physical independence and daily activities. Similarly, the Neuropsychology/Psychology team may concentrate on cognitive and emotional rehabilitation for individuals with neurological conditions, requiring a deeper focus on psychological and brain function assessments. Meanwhile, the Pediatrics team includes multiple disciplines, working with children whose rehabilitation needs often overlap with developmental milestones, necessitating integrated approaches across therapies. Given these variations, some decisions and methodologies may differ between teams to accommodate their respective client populations and objectives. This flexibility allows each team to tailor their approach to their specific field while adhering to shared overarching principles. The coordinator’s role is to mitigate this limitation by tracking the decisions made by each team, ensuring consistency where necessary, and allowing each group the flexibility to best conduct the review in its field.

## 5. Conclusions

This series of rapid reviews will synthesize the extensive body of evidence, including studies conducted in real-world settings, on telerehabilitation. The reviews will explore its strengths and weaknesses, assess its ethical implications and examine how equity issues can be identified and addressed to guide the continued implementation and use of telerehabilitation. By analyzing the impact of telerehabilitation on various patient populations and on professional practices, the findings will provide actionable insights to inform future guidelines and standards. These insights have the potential to influence telerehabilitation policies and practices; ensuring that services are accessible, effective, ethical and equitable. Moreover, the recommendations derived from this work can support policymakers, healthcare providers and stakeholders in addressing systemic barriers, promoting ethical and equitable practices. It could foster innovation in service delivery. Additionally, the recommendations derived from this work can support a better integration of telerehabilitation into educational curricula, professional training programs, and continuing education initiatives. Ultimately, this effort aims to contribute to a more inclusive and evidence-based telerehabilitation framework that meets the needs of diverse communities.

## Figures and Tables

**Table 1:** Criteria for inclusion/exclusion of studies.

Criteria		
PICOTSS	Inclusion	Exclusion
Population	Patients of all ages who had telerehabilitation at least once in at least one of those rehabilitation fields: physiotherapy, occupational therapy, speech therapy, audiology, neuropsychology, and psychology.	Animal studies will be excluded.
Intervention	Any telerehabilitation interventions in at least one of those rehabilitation fields: physiotherapy, occupational therapy, speech therapy, audiology, neuropsychology, psychology. This includes synchronous, asynchronous and hybrid interventions (e.g., virtual assessments/treatments, recorded videos, phone calls, videoconferencing, and mobile applications if there is an input from a clinician).	Studies that mention smart watches/activity trackers, and mobile applications without the implication of a clinician are excluded (e.g., no meetings to discuss the results, no feedback provided by a clinician).
Comparator	Not applicable	
Outcomes	Any outcome included in The International Classification of Functioning, Disability and Health (ICF): efficacy, engagement, acceptance, feasibility, patient satisfaction with the technology, health services outcomes, equity and ethical outcomes (e.g., technology access/cost, issues of consent, among others.)	Outcomes not relevant to the scope of the review, such as weight gain/loss, etc.
Timing	Studies from January 2010 to March 2023. More specifically, review of reviews from 2010 to 2020, and individual articles from 2020 to 2023 are included.	
Settings	All setting will be included: (e.g., home, nursing home, long term care).	No exclusion applied as long as telerehabilitation services are being provided.
Study design and other limiters	Systematic reviews, rapid reviews, meta-analysis, scoping reviews or narrative reviews.	Protocols, case report or case study, literature or narrative reviews that did not include a systematic search and description of each study included will be excluded.

## Data Availability

The raw data supporting the conclusions of this article will be made available by the authors on request.
